# Identifying coping strategies used by patients at a transgender health clinic through analysis of free‐text autobiographical narratives

**DOI:** 10.1111/hex.13222

**Published:** 2021-02-24

**Authors:** Angela Zottola, Lucy Jones, Alison Pilnick, Louise Mullany, Walter Pierre Bouman, Jon Arcelus

**Affiliations:** ^1^ School of English University of Nottingham University Park, Nottingham United Kingdom; ^2^ School of Sociology and Social Policy University of Nottingham University Park, Nottingham United Kingdom; ^3^ Nottingham National Centre for Transgender Health Nottingham United Kingdom; ^4^ Institute of Mental Health University of Nottingham Nottingham United Kingdom; ^5^ Present address: Dipartimento di Culture, Politica e Società Università degli Studi di Torino Torino Italy

**Keywords:** communication, coping strategies, empowerment, secondary care, transgender persons

## Abstract

**Background:**

This paper presents an analysis of 32 narratives written by patients waiting for assessment at a transgender health clinic (THC) in England. Narratives are autobiographical free texts, designed to allow patients to describe in their own words their experiences of their gender identity and/or transition prior to a clinic appointment, as part of the assessment process.

**Objective:**

Narratives were analysed to identify actions prospective patients had taken to manage their (usually lengthy) waiting times, so that these ‘coping strategies’ could be shared with future patients.

**Design:**

Corpus linguistic methodology was utilized to identify common patterns across the whole corpus of text‐based data, augmented with more detailed sociolinguistic analysis of individual narratives.

**Results:**

There are broad commonalities in the way the transition experience is described across the corpus in terms of presentation of key experiences and feelings. There are specific descriptions of a number of recurring coping strategies, both positive and negative.

**Conclusion:**

The empowerment value of writing these narratives may be limited; the existence of recurring key features suggests that patients may feel they have to present their experiences in certain ways to be accepted for treatment. However, dissemination of some positive coping strategies may help future clients of THCs to better cope with waiting times, as well as assisting practitioners in THCs in supporting their patients during this wait.

**Patient/Public Contribution:**

The clinic's Service Users’ Research Advisory Group contributed to formulating the objective and design of the study. Results were presented at the clinic's annual PPI conference.

## 1 INTRODUCTION

This paper presents an analysis of 32 narratives written by patients waiting for initial assessment at a transgender health clinic (THC) in England, between 2006 and 2016. Patients were asked by clinicians to produce these narratives to describe their experiences of gender identity and/or transition, from whichever point in their life they chose to begin until the time of writing. Clinicians at the THC designed the writing of these free‐text narratives to empower patients to tell their own stories, in their own words; these stories were then used as part of the initial assessment process by the clinic. Using corpus linguistic techniques augmented by a more detailed sociolinguistic analysis, the aim of this study was to identify the actions patients describe using to manage their day‐to‐day lives prior to referral and to consider the value these actions had for the patients and how this knowledge could be of use for future patients. Some of these actions are described as having a positive impact, and others, a negative impact. Information on these positive and negative ‘coping strategies’ is potentially useful both for future patients and for practitioners in supporting these patients. The ultimate aim of the study was to facilitate positive strategies to be disseminated more widely where appropriate and to enable patients to be supported in avoiding or managing more negative approaches.

## 2 BACKGROUND

The social, political and medical climate in relation to transgender identities is constantly evolving, and this is reflected in changes to diagnostic criteria. How a condition is defined and diagnosed not only has political and social ramifications but, importantly, affects how people are perceived by others and how people perceive themselves. The two prevailing classification systems in this field are the World Health Organization's (WHO; 1990) International Statistical Classification of Diseases and Related Health Problems (ICD) and the American Psychiatric Association's (APA) Diagnostic and Statistical Manual of Mental Disorders (DSM).^1^ Unfortunately, these classification systems are not revised simultaneously and, so far, have not reached a consensus. During the period in which the patients in our study were referred to the clinic, the ICD classifications remained constant, but there was significant change to the DSM classifications in 2013. ICD classifications were then revised in 2018. We give a brief overview of these changes below, since they are relevant for understanding the environment within which patients produce the narratives analysed here.

At the time the patients in our study were referred to the transgender health clinic (THC), and until 2018, for an adult to meet the criteria for a diagnosis of Transsexualism (F64.0), according to the International Classification of Diseases version 10 (ICD‐10), they had to have expressed the desire to live and be accepted as a member of the opposite sex, usually accompanied by the wish to make their body as congruent as possible with the desired sex through surgery and cross‐sex hormones. This trans identity must have been present persistently for a minimum of two years and not be a symptom of another mental disorder or a chromosomal abnormality.^2^ Transsexualism was categorized in ICD‐10 as a mental health condition. However, it is worth noting here that, with the production of ICD‐11 in 2018, the official terminology was revised, alongside the classification of the condition. ‘Transsexualism’ was replaced by the term ‘gender incongruence’, where this is defined as ‘a pronounced, persistent incongruence between the individual's experience of gender and the sex assigned’.^3^ Gender incongruence is now categorized as a condition related to sexual health.^4^

In terms of DSM categorizations, the term transsexualism was replaced with ‘gender identity disorder’ in DSM‐IV^5^ and classified as part of a broader category of sexual disorders. In the DSM definition, gender identity disorder is seen as having two components: a strong and persistent cross‐gender identification, and a sense of persistent discomfort with one's sex. Midway through the period in which the narratives for this study were written, the publication of DSM‐5 (2013) introduced the specific term ‘gender dysphoria’; this term assigns the pathology of the condition within the dysphoria, as opposed to within the gender identity.^6^, ^7^ The distress intrinsic to gender dysphoria may be focused around anatomy, physiology, and/or being perceived and treated as someone of a sex with which the person does not identify.^8^ This distress may lead individuals to transition, so this mismatch is no longer present; most commonly transitioning from a man to a woman (people known as trans women) or from a woman to a man (people known as trans men).^9^, ^10^ However, these diagnostic labels do not apply to all transgender individuals, as some people will not identify themselves as a man *or* as a woman.^10^, ^11^, ^12^

In the UK context, patients diagnosed with transsexualism (pre‐2018) or gender dysphoria are able to access gender‐affirming medical treatments through THCs, including the prescription of cross‐sex hormones, voice coaching, referral for facial hair removal and psychological support. After a minimum of one year living in their acquired social gender role and taking cross‐sex hormones, patients, if they so wish, may also be referred for gender‐affirming surgeries.

The process outlined above is far from uncontroversial. Some scholars and activists have argued against the provision of specialist services through THCs on the basis that they represent the medicalization of trans identity, and are institutions acting as gatekeepers to trans people's self‐fulfilment through gender‐affirming surgery.^13^, ^14^ However, other scholars have noted that medicalization does not have a unidirectional impact, and that current frameworks legitimize gender transition as a process that is medically necessary for an individual, and so support a pathway to gender‐affirming care for those who need it.^15^ One area where there is general consensus, however, is concern over the waiting time to be seen by a THC in the UK. At the clinic where this study was carried out, in 2016 average waiting time from referral to first appointment was 6 months, but by 2020, this had risen to more than 3 years. This increase does not only reflect the increased number of referrals, but more specifically chronic underfunding for transgender health care.^16^ The issue of how patients can be supported to manage these waiting times is therefore of growing importance.

## 3 METHODS

Data for this study come from patients attending (the Nottingham Centre for Transgender Health), a THC forming part of the national service for people living in England who present with unhappiness, discomfort and/or distress about their sex observed at birth. Patients are primarily referred by general practitioners (family doctors or physicians). The assessment process usually requires two appointments with two different clinicians, followed by a third meeting with both clinicians, the trans individual, and someone who is close to them and has known them for some years. As part of the assessment process, the trans individual is required to provide evidence of social gender role change, including their change of name by deed poll or statutory declaration, plus a minimum of two official documents such as passport and driving license in their changed name. Following this, the two independent transgender health specialists reach a diagnosis. Until 2016, patients were also invited to write an autobiographical narrative of their ‘gender journey’ which had brought them to the clinic, to be submitted prior to the third meeting in hard copy. Patients were simply invited to write down their life histories, from their own perspectives—no specifications on format or style were given. The process of writing these narratives was seen by the clinicians as empowering, since patients themselves controlled the style and content. It was also designed to be both therapeutic and informative, assisting clinicians in reaching a diagnosis and later deciding on the treatment options most appropriate for the patient. Due to a change in practice, narratives are no longer routinely collected by the clinic for diagnostic purposes. However, what they tell us about how prospective patients manage their wait for referral is still a useful resource, with on‐going relevance for future patients who also face this wait.

Before we began the study, an initial proposal was submitted to the Service Users Research Advisory Group at the THC. Following their discussion and approval, UK National Health Service ethical approval was granted in autumn 2018 (reference 18/LO/0708). One condition of this approval was that patients should only be alerted to the study and invited to participate by their clinicians when they were on‐site for appointments. This limited the number of narratives that could be included, since many authors of earlier narratives were no longer attending the clinic. In total, we gained consent from 32 participants to use their narratives; due to patient confidentiality and the anonymity embedded into our research design, we are unaware of the number of patients who were actually approached to participate.

**FIGURE 1:**
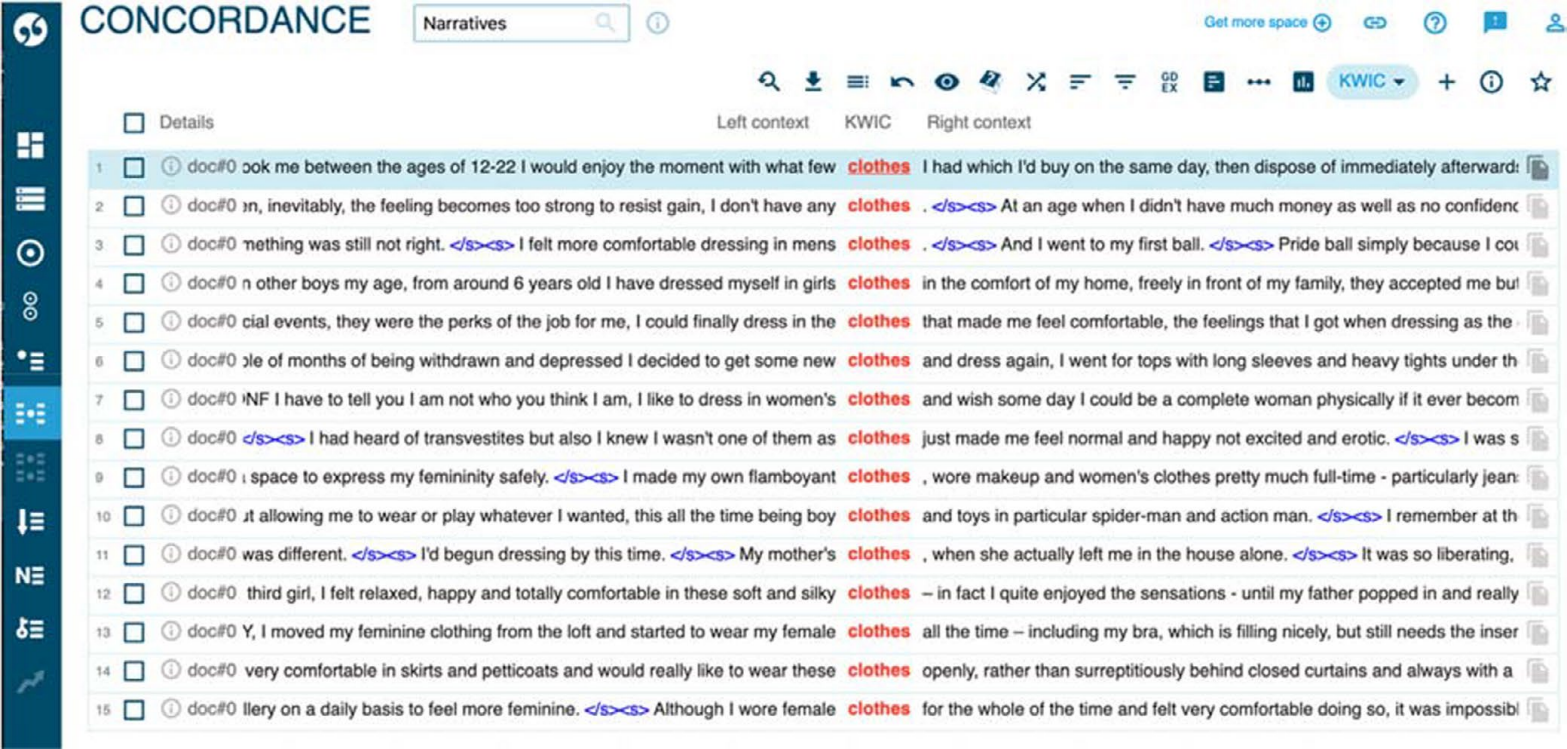
Concordance list of ‘clothes’ from Sketch Engine

Narratives were anonymized and transcribed, with pseudonyms assigned to the participants. Basic demographic information such as age, ethnicity and religion, as well as the time between the referral and the first appointment, was also noted on the file. To prepare the anonymized files for use in corpus linguistic software, each transcription was saved as a.txt file and encoded using XML.^17^ Of the 32 texts collected, 17 were produced by trans men and 15 were from trans women, all identifying as white and British, and born between 1943 and 1998. Because we were unable to access records about the typical demographics of the clinic's patients, we cannot say whether this was a representative sample in terms of nationality and ethnicity. With a more diverse range of participants, we may have been able to gather additional intersectional insights into the experiences of those accessing the clinic. As shown in Figure 1, most trans women in our corpus were older at time of writing (40+ years), while the trans men were younger (16+ years).

Trans women have been represented more than trans men in previous research,^9^, ^10^ but in recent years there has been a significant rise in the number of trans men referred to THCs.^18^ One pattern apparent in our data set prior to analysis was the length of the narratives; the trans men typically wrote much less (totalling 15,766 words from 17 participants), while the trans women wrote much more (totalling 80 289 words from 15 participants). However, narratives were commonly structured in chronological order, from childhood through to adulthood, and so rather than reflecting some inherent gender difference between our participants, it seems likely that the trans women in our sample—with an older age profile—had more life‐experience to record (Table 1).

**TABLE 1:** Age of participants

Decade in which the participants were born	Trans women	Trans men
1990	1	14
1980	0	1
1970	6	0
1960	3	2
1950	3	0
1940	2	0

**FIGURE 2:**
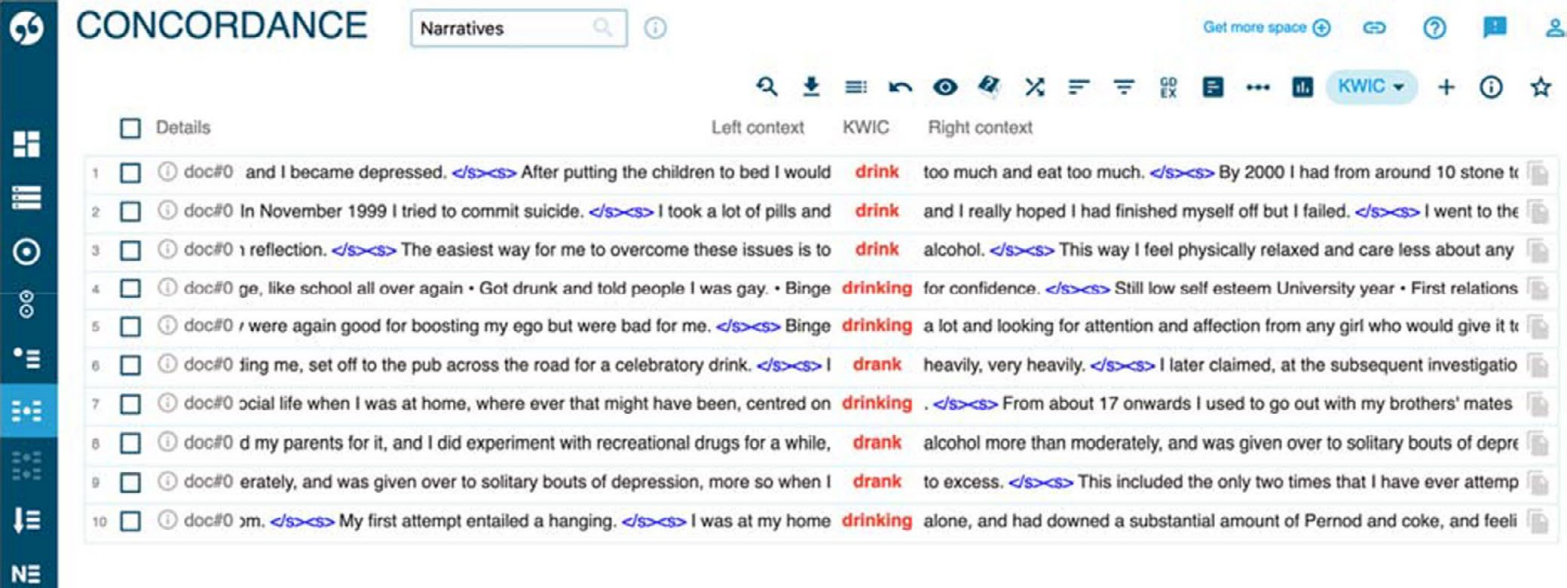
Concordance list of ‘drink*’ from Sketch Engine

**FIGURE 3:**
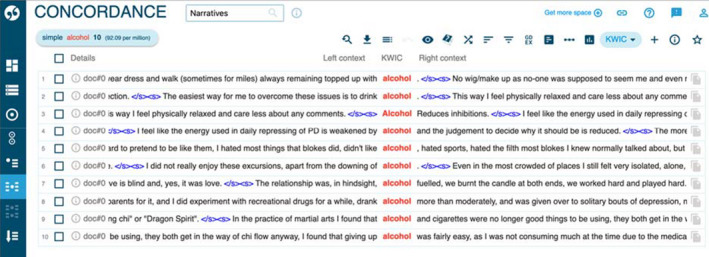
Concordance list of ‘alcohol’ from Sketch Engine

Our corpus comprises 96,055 words and is divided into two subcorpora: narratives from trans men and narratives from trans women. We used Sketch Engine^19^ to investigate our corpus, following Tognini‐Bonelli^20^ and O’Keefe.^21^ Corpus linguistics is a computer‐mediated approach that allows the analysis of large amounts of data through the use of software that can search for specific words in given combinations. This approach was used in combination with sociolinguistic analysis. Within this framework, scholars study the relationship between language and society, with a particular focus on identifying the social function of language and how this is used to convey social meaning.^22^ For large corpora consisting of millions of words, corpus linguistics is used to identify quantitative patterns; more detailed qualitative analysis is often then used to closely examine instances of the patterns identified. For smaller corpora, as here, this process is reversed: we began with close sociolinguistic analysis of the texts to identify how salient features related to coping were expressed and then applied the corpus linguistic technique of ‘concordance analysis’ to identify and map the presence of these features across the whole corpus. The technique involves the creation of a concordance list, ‘[…] a list of all the occurrences of a particular search term in a corpus, presented within the context they occur in’.^23^ In fact, once the concordance list is generated each line presents the search term within the sentence in which it is contained, and it also allows the researcher to access the rest of the narrative by clicking on the term to investigate the context even further (see, eg, Figures 1, 2 and 3). This ensures that the word or phrase that is being analysed is not considered out of or disembodied from the context in which it is produced. Thanks to this function the researcher is able to establish the type of evaluation carried by the linguistic unit being analysed. This combination of approaches allowed us to produce an overview of the coping strategies laid out in our participants’ narratives and also to examine contextual variation in these.

Because ethical approval conditions meant we dealt only with anonymized data, we were unable to engage in conventional member checking work to verify our analysis with the authors of the narrative. However, we did discuss our findings with a group of current service users at the end of the project and received positive feedback as to the accuracy of our analysis. Additionally, as a way to enhance reliability, all authors in our interdisciplinary team contributed to the analysis, thereby ensuring linguistic, sociological and medical perspectives were included.

## 4 RESULTS

Despite the demographic differences within our corpus, we identified a number of commonalities to the narratives. Participants usually described feeling different from early childhood. This led to an early rejection of gender stereotypes associated with their sex category and reported distress when they were required to conform to these stereotypes. By contrast, adoption of opposite gender stereotypes (eg appearance and clothing) was described as alleviating distress. Reports of attempts to change or deny feelings around gender identity were also common, and always presented as ultimately unsuccessful. More specifically, our linguistic analysis identified a number of actions that participants reported taking during the period before referral to the clinic. These actions were explicitly presented by participants as everyday ways of managing emotions and experiences related to the mismatch between their gender identity and their sex, though the categorization ‘coping strategies’ is an analytic one since participants did not necessarily use that term. The coping strategies were grouped into two main categories: those presented as positive actions by our participants and those presented as negative. Clearly, these are their own subjective evaluations but, in their narratives, participants framed these actions not only in their own terms but also in terms of the consequences they alleviated or prevented. For example, purchasing and taking non‐prescribed hormones resulted in physical change which participants reported as alleviating their dysphoria, and which they therefore saw as outweighing any risk to their physical or emotional wellbeing. Table 2 shows the occurrences of each strategy in the corpus, as established through frequency counts and concordance analysis.

**TABLE 2:** Coping strategies

Positive Strategy	occurrences	Negative Strategy	Occurrences
Dressing	59	Alcohol abuse	37
Hormones	13	Self‐harm	28
Support [friends‐specialists‐groups]	13	Denial of gender identity	22
Online persona	10	Drug abuse	3
Coming out	4	Food abuse	3
Research	4		
Prayer	1		

### 4.1 Positive strategies

Of the coping strategies classified by participants as positive, some are material (eg dressing, hormone taking) while others are non‐material (eg creating and inhabiting an online persona, carrying out research). Extract 2 in Appendix A represents an example of the most frequent material positive coping strategy, ‘dressing’.

In presenting this as a positive strategy, the participants generally describe the practice of wearing clothes, hairstyles or accessories that are stereotypically representative of the gender they identify with, and the experience is then associated with positive affect, demonstrated through the narratee's positive evaluative lexis, for example ‘it felt kind of wonderful’. A snapshot of representative concordance lines related to ‘dressing’ is shown in Figure 1 (‘clothes’ are part of the overall ‘dressing’ strategy). By observing the figure, it is possible to note some of the other occurrences of affect in the corpus; in the third concordance line for example the participant affirms that ‘I felt more comfortable dressing in men's clothes’. Similarly, the participants express being comfortable with using clothes of their identifying gender in the fifth concordance, ‘I could finally dress in the clothes that made me feel comfortable’.

Another material strategy our participants described was obtaining and taking cross‐sex hormones. In some cases, participants obtained these from medical professionals outside the UK; in other cases, as in Extract 3 in Appendix A, these were purchased online.

The instances in which the participants discuss taking hormonal treatment, as in Extract 1 and Extract 3 in Appendix A, are always followed by descriptions of a sense of happiness and fulfilment. Extract four in Appendix A is a very explicit example of this feeling of happiness that comes with taking hormones. Extract three also shows that Anne describes seeking hormones when the material strategy of dressing became insufficient on its own to alleviate feelings of depression.

The most common non‐material positive strategy described by participants is the use of an online persona, which was described as a means to freely express their identity in a safe space without fear of being judged, insulted or harassed. Extract 5 in Appendix A is an example of this.

The use of an online persona was commonly presented as a moment of liberation and freedom. Importantly, for some of our participants, it was also presented as a strategy with potential advantages over material strategies such as dressing, as there was less fear of being discovered. Extract 6 and Extract 7 show how this is described by our participants. Mary specifies that online ‘no‐one ever knew’ while John stresses how he ‘could be whoever [he] wanted to be on the internet’.

Less frequent in our data, but also relevant for this analysis, is the presence of examples of coming out stories; these were categorized as coping strategies because in our corpus they are always followed by the author positively describing a sense of relief that this action gave them, or how it made them feel empowered to take further action, as Extract 8 in Appendix A shows.

Extract 8 presents one of the two different versions of a ‘coming out’ narrative present in the data: in this case, the coming out is private, to a single, trusted friend, whereas other examples show a more public ‘coming out’, in the workplace, for example, or on social media, see Extract 9 in Appendix A. However, in both versions the end result is presented as a means to achieving a greater degree of self‐acceptance.

Another action presented positively by our participants and which we categorized as a coping strategy was that of conducting research. This was usually carried out online, browsing the web in search of ways of mitigating negative feelings. Extract 10 in Appendix A is an example of this.

As the extract shows, participants reported their searches leading to a realization that others felt the same way as they did, or the discovery that definitions others had applied to themselves fitted their own experiences. These narratives often contain phrases describing a specific moment of realization, such as ‘clicked in my head’ (as above) or ‘I knew that it related to me’.

### 4.2 Negative strategies

Many of the positive strategies described above represent ways in which our participants were able to embrace their gender identity. However, most participants also reported initial attempts to cope through denial. Extract 11 in Appendix A illustrates an attempt at denial as a coping strategy, which was ultimately unsuccessful.

Extract 11 also illustrates another common phenomenon in our data; that denial was accompanied by an attempt to embrace a material strategy to reinforce this denial, such as the adoption of a hobby or behaviour stereotypically associated with birth sex. Such attempts were always presented as short lived.

Other actions that were presented involved destruction of the body in some way, using alcohol, food, drugs or self‐harm. Figures 2 and 3 show frequent occurrences of participants describing alcohol abuse, reporting using large amounts of alcohol (ten instances), or the verb ‘to drink’ (27 instances) when describing their actions.

The use of drugs/substance abuse (as in the second concordance line in Figure 2) is discussed less frequently than the abuse of alcohol, and the practice of overeating is also found in three examples, as Extract 12 in Appendix A illustrates.

These examples show the context in which these behaviours are presented in the narratives, where they are used as everyday strategies to fight depression or as a way to cope with unhappiness but are also recognized as having a negative impact. Other narratives show similar descriptions of cannabis use, see Extract 13 in Appendix A. It is noteworthy, however, that for 27 out of 32 of the participants in our corpus, narratives move at some point beyond ‘everyday’ harms to discussion of suicidal thoughts or even attempted suicide as a way to manage negative experiences. Extract 14 in Appendix A illustrates this (another example is found in the second concordance line of Figure 2).

As in Extract 14, in our corpus participants generally presented suicidal thoughts as arising in response to a specific incident or occurrence, in contrast with ‘everyday’ strategies relating to food, drugs or alcohol.

## 5 DISCUSSION AND CONCLUSION

### 5.1 Discussion

This study draws on data emerging from a corpus of 32 narratives. The necessary constraints of our research design meant we were unable to obtain more data; this is a limitation of our study. However, we have demonstrated here clear patterns in the data, indicating the representativeness of our sample. Furthermore, our dissemination to the clinic's service users revealed that they felt the data obtained gave an accurate reflection of their collective experiences.

Reflecting first on the positive strategies presented in our data, there is evidence of an individual developing an external representation of themselves which allows them to be seen as ‘authentic’ regarding their felt gender. Non‐material strategies, such as online research, are often presented as a precursor to material strategies of dressing or hormone taking. In this way, it can be argued that embodiment acts as a semiotic practice^24^ and that our participants’ narratives show the importance of them *both* feeling authentic and being perceived as authentic by others. It could therefore be argued that trans people seeking treatment are aware of a need to conform to a normative ideal to ‘pass’ as their felt gender: our data show the ways in which stereotypically ‘male’ or ‘female’ appearances and activities are embraced or rejected in order to support this passing. However, as Edelman^25^ argues, this kind of binary gender productivity is likely to be reinforced by a system which requires successful passing as a pre‐requisite to medical treatment.

An institutional framing also arguably exists in the accounts of negative strategies. Given the gatekeeping function of THC referrals,^13^, ^14^ patients may believe they are required to articulate these negative experiences in order to match the criteria for being treated; in other words, they may want to emphasize the difficulties they face to emphasize their need for NHS services. In this sense, it may be the case that these participants emphasized negative experiences in order to seem legitimate. On the other hand, many trans people going through this process will be aware that being seen to have poor mental health could work against them in securing a diagnosis of gender dysphoria, which may create an incentive to downplay some aspects of their lives. Johnson^15^ has highlighted the way in which a number of studies of trans people's experiences^26^, ^27^, ^28^ demonstrate their nuanced and strategic use of diagnostic and medical logics in order to access support, and we recognize that the specific context in which these narratives were produced is likely to result in a particular telling of people's positive and negative experiences.

This institutional framing also has implications for the empowerment value of the narratives. As Prosser^29^ notes, the diagnostic situation provides a place for an individual to tell and begin to realize their story. However, the rationale for allowing patients to tell their own story in their own words is undermined if they feel that they must nonetheless follow a particular script. The clinic ceased collecting the narratives in 2016 because they were no longer felt to be useful in the diagnostic process, and our analysis here sheds some light on why this may be so; this analysis chimes with prior work eliciting care providers’ views which highlights the awareness of a need to ‘work the system’.^29^ Nonetheless, patients’ descriptions of the day‐to‐day management of their own lives while they await diagnosis and treatment still represent a valuable resource, and Dewey's^30^ work also highlights the desire of care providers to be better equipped to provide support.

### 5.2 Conclusion

Because the narratives in our corpus are likely framed by the institutionalized gatekeeping role of the THC, it is feasible that there are additional strategies not detailed by participants for fear of appearing inauthentic, or that some experiences are deliberately emphasized over others. Of course, our data are restricted in this way. However, the patterns identified within them nonetheless provide useful insight into some commonly held experiences shared by patients while waiting for treatment at the clinic. We therefore offer here a summary of these insights and some recommendations for practitioners.

Our analysis shows trans people report that representing their gender identity externally can be an important factor in their well‐being. We therefore suggest that THCs offer more advice to future patients on how to do this. We also note that many of our participants report that they began their transition journeys by using non‐material strategies such as online research or the use of an online persona, before they were ready or able to move to material strategies such as dressing. Signposting to online resources and suggesting the adoption of a virtual persona are other avenues of support that could be offered by THCs. Use of cross‐sex hormones prior to treatment by the clinic was commonly presented as a positive strategy in our data, with these being obtained either from professionals outside the UK, or online. However, this has associated and potentially negative implications for physical health, and so, acknowledgement of the possibility of this strategy is important for practitioners in advising patients.

An identification of the negatively characterized strategies presented here offers an opportunity for THCs to signpost to other relevant services where support is available, for those dealing with issues related to food, alcohol or drugs. Identification of these coping strategies also offers the opportunity for development of more targeted service provision in the future, to assist both in the uptake of positive coping strategies and the management of negative ones. These findings will thus be of practical use to those individuals who are waiting to be assessed at a THC, for clinicians who work there and for medical educators.

## CONFLICT OF INTEREST

There are no conflicts of interest to declare for any of the authors.

## AUTHORS CONTRIBUTION

AZ with support from all authors carried out data collection, designed the analytical approach and prepared a first draft of the analysis. LJ was PI for the project and, with WB and JA, designed the data collection and gained ethical approval for the project. AZ, LJ, LM and AP produced the analysis, and AP prepared a first draft of this manuscript. All authors made substantial contributions to the revising of the manuscript and approved the final version.

## Data Availability

The data referred to in this manuscript come from a corpus. A condition of ethical approval from our NHS Research Ethics application was that only isolated quotes would be used in published material and that the corpus as a whole will be destroyed after 10 years. We therefore cannot share the entire corpus.

## APPENDIX A

### Extract 1: John

The first night I arrived in Thailand I was treated as a man. […] I was being seen for who I really was for the first time in my life. And I loved it! I knew then that this was who I am and how I wanted to be treated as a man. I knew when I came home I would never be the same person again. I would never allow people to tell me what I should wear just because they thought I should or how I should feel. But at the same time I knew it was not going to be easy for people especially those who had known me for years. Thailand is where I started on Androgel and I was never as happy in all my life as when i got the go ahead for Androgel from an endocrinology doctor in Thailand. I did not want to come home to rejection from my own doctor at home.

### Extract 2: Carly

I never told him or Francis [Carly's relative] that I was trying her clothes on occasionally too and that it felt kind of wonderful.

### Extract 3: Anne

[…] I also started to get really depressed, I had not ever been depressed before when I was dressed, it had always helped in the past to alleviate the depression, I needed more, so I decided that I would try hormones first to see if they helped me, I had been chatting online to some other trans girls and a few had said that hormones had helped them, I went online and got 6 months of hormones, started taking them 17th May the first 2 weeks I was quite disappointed as nothing seemed to be happening, then about 3 weeks on hormones I started to feel really happy, confident and the depression was gone, even though I was still devastated at the loss of my wife, but I felt like a new person, it was incredible, I was more confident in myself than I had ever been, even when dressed as Anne.

### Extract 4: Carly

Starting hormones two years ago and just being myself has opened my emotions up to a rainbow of colours where all I ever aimed for was contentment, a quiet place in which survival might remain possible. I feel joy, for the first time ever in my life, I feel joy. Can you imagine what feeling joy for the first time has been like for me? I never knew it before and I love it.

### Extract 5: John

For a while I had a Facebook that was fake so I could get treated like a boy. I enjoyed role‐playing as male characters online, and it served as an outlet for my dysphoria.

### Extract 6: Mary

The online presence started in 2001 via Yahoo pool which was nice to play as a girl and without pictures or other methods of contact no‐one ever knew.

### Extract 7: John

In the meanwhile I spent a good few years on a game site called its your tum on my pc. […] It was on this game site that I first used my male gender. Because I could be whoever I wanted to be on the internet.

### Extract 8: Beth

I pushed him away and it got me thinking I was gay. I confided in a close friend at work and she was quite surprised that I thought I was gay. Then she said she thought I would have been more into cross dressing. At Christmas I confessed to her that I wanted to be a woman and told her about how I felt. This conversation made me determined to try and sort myself out.

### Extract 9: Roxanne

The date was the 21st January 2012. This was the day I officially started work as Roxanne. God, I was so nervous before and when I entered work. I had applied a little makeup and changed into my female uniform. How would people react?

I had previously arranged with my supervisor to make a statement to all the staff (about 30 in all) at the start of the shift. After completing a number of house‐keeping matters, she (my supervisor) said that Roxanne would like to share something with you all. I stood up, shaking like the preverbal leaf, and read out, with a few embellishments, the following statement: […]

Immediately afterwards, I was hugged by two of the sisters with comments of support. This continued throughout the day; hugs, handshakes and messages of support from supervisors, staff and colleagues. One staff member said it was the "gutsiest" thing he'd ever seen in his life. Somehow, I felt so relaxed and calm and at one with myself. It was probably my best day at work—ever. The first day of the rest of my life.

### Extract 10: Greg

Near the end of counselling I remember on the internet I read a list of a person being depressed and how he realised he was transgender. This clicked in my head and I realised who I was, counselling soon ended an I got referred to CITY GIC.

### Extract 11: Kate

The sense of shame and disgust, however, got stronger with time and I would periodically throw out all of the women's clothes I had (which were usually stolen because I felt so much shame and fear at the thought of buying them myself) and force myself into something masculine like weight training. It never lasted more than a few months.

### Extract 12: Beth

I had no time to do what I wanted and I became depressed. After putting the children to bed I would drink too much and eat too much. By 2000 I had from around 10 stone to 15 stone.

### Extract 13: Lucy

I phoned a helpline get chatting—that when I started using PFF as my name went out a few times, but at the time I was bit depressed and was smoking cannabis I thought I was too tall so ended up loosing contact but carried on dressing at home in secret.

### Extract 14: Beth

That was my first thought of suicide, after being caught in girls’ undies all I wanted to do was kill myself. I spent most of the next day on a motorway bridge but I could not jump.
